# The Selective Interaction of *Pistacia lentiscus* Oil vs. Human Streptococci, an Old Functional Food Revisited with New Tools

**DOI:** 10.3389/fmicb.2017.02067

**Published:** 2017-10-24

**Authors:** Germano Orrù, Cristina Demontis, Antonello Mameli, Enrica Tuveri, Pierpaolo Coni, Giuseppina Pichiri, Ferdinando Coghe, Antonella Rosa, Paola Rossi, Guy D’hallewin

**Affiliations:** ^1^Molecular Biology Service, Department of Surgical Sciences, University of Cagliari, Cagliari, Italy; ^2^Department of Surgical Sciences, University of Cagliari, Cagliari, Italy; ^3^University Hospital Laboratory Services, Azienda Ospedaliero-Universitaria di Cagliari, Cagliari, Italy; ^4^Department of Biomedical Sciences, University of Cagliari, Cagliari, Italy; ^5^Department of Biology and Biotechnology “L. Spallanzani”, University of Pavia, Pavia, Italy; ^6^National Research Council of Italy, Sassari, Italy

**Keywords:** *Streptococcus* spp., *Pistacia lentiscus* oil, natural antimicrobials, free fatty acids, biofilm inhibition

## Abstract

*Pistacia lentiscus* berry oil (LBO) represents a typical vegetal product of the Mediterranean basin that has been formally used in traditional cuisine for 100s of years. In addition to its interesting alimentary properties, this product could represent an interesting candidate in the field of research on the study of new anti-infective agents. In fact, in Mediterranean countries, lentisk oil still continues to be widely used in folk medicine for oral and skin affections, in particular, acute gingivitis, pediatric skin infections such as impetigo and foot plaques, and biofilm related infections often associated with *Streptococcus* spp. Following these observations, we have hypothesized a “lentisk oil-bacteria” interaction, placing particular emphasis on the different Streptococcal species involved in these oral and skin diseases. In accordance with this hypothesis, the use of standard antimicrobial-antibiofilm methods (MIC, MBC, MBIC) allowed the interesting behavior of these bacteria to be observed and, in this context, the response to lentisk oil appears to be correlated with the pathogenic profile of the considered microorganism. Two probiotic strains of *S. salivarius* K12/M18 appeared to be non-sensitive to this product, while a set of five different pathogenic strains (*S. agalactiae, S. intermedius, S. mitis, S. mutans, S. pyogenes*) showed a response that was correlated to the fatty acid metabolic pathway of the considered species. In fact, at different times of bacteria development, selective High Performance Liquid Chromatography analysis of the growth medium containing LBO detected a significant increase in free unsaturated fatty acids (UFAs) in particular oleic, palmitic and linoleic acids, which are already known for their antibacterial activity. In this context, we have hypothesized that LBO could be able to modulate the pathogen/probiotic rate in a Streptococcal population using the fatty acid metabolic pathway to help the probiotic strain. This hypothesis was strengthened by performing antibacterial testing with oleic acid and an *in silico* evaluation of the Streptococcal MCRA protein, an enzyme involved in the production of saturated fatty acids from UFA. These results show that LBO may have been used in ancient times as a “natural microbial modulating extract” in the prevention of biofilm- associated diseases.

## Introduction

The current emergency of antibiotic resistance poses a serious problem for human health as regards the treatment of various bacterial infections. Epidemiological reports by numerous clinical researchers or national associations have specified the seriousness of the situation and, as proof of concept, research has been focusing on new therapeutic strategies for novel antimicrobials and also showing interest in bioactive molecules extracted from plants or fruits (Pogany Simonova et al., 2009; Verkaik et al., 2011). To date, the antibacterial proprieties/mechanisms of foodstuffs or food components, particularly those of ethnobotanical use, are unknown or barely known (Reid, 2000). Although this approach could be promising, different questions need to be addressed. For example, many plant extracts could be toxic for human tissues, and their safety assessment tests are time consuming and expensive (Chanda and Baravalia, 2011; Coe et al., 2012). A further problem is that many human infections are supported by a strong biofilm, which adheres to the tissue surface providing a protective coating that is impermeable to antimicrobials. Such behavior has been described in various body regions, e.g., in the oral cavity and on the skin (Beikler and Flemmig, 2011; Vieira Colombo et al., 2015). The biofilm produced by the bacteria is crucial for virulence potential as compared to its planktonic counterpart (Marks et al., 2014). In fact, this sessile *status* has several advantages for a pathogen, including antimicrobic resistance due to lack of drug delivery and delay of bacterium recognition by the host-innate immune system (Bahar, 2002). A solution to this biological/medical problem could be a new approach, able to decrease the populations of pathogen bacteria in the biofilm and, subsequently, target host response especially in the first steps of the infection (modulating activity). In this work, we studied the anti-microbial motif of an oil obtained from the fruit of an ethnobotanical plant that was first used 5,000 years ago by the Bronze Age Paleolithic communities of several Mediterranean and Middle Eastern countries (Bozorgi et al., 2013). This shrub, namely lentisk (*Pistacia lentiscus* L.), belongs to the *Anacardiaceae* family and almost the whole plant has been used in traditional medicine. Most research has focused on the antimicrobial properties of the non-edible part of this plant, i.e., mastic gum or essential oil, obtained from the resin and branches/leaves, respectively. Each part shows different proprieties: mastic gum (produced on the island of Chios) is active against *Helicobacter pylori* infection, while the leaf-essential oil affects different bacterial species including some oral pathogens such as: *Porphyromonas gingivalis, Streptococcus mutans, Streptococcus gordonii, Fusobacterium nucleatum, Prevotella intermedia* (Pham-Huy et al., 1999; Koutsoudaki et al., 2005; Aksoy et al., 2006). The oil produced by pressing the berries represents the liquid fraction at room temperature and the antimicrobial activity of lentisk oil obtained from the berries (LBO) on *Staphylococcus aureus* and *Aspergillus niger* it has recently been reported (Mezni et al., 2014a, 2016). At present, few studies exist on the interactions of LBO with microorganisms and on its antimicrobial mode of action. In the present work, the activity of this edible oil was investigated on a set of microbes that are characterized by a different typology of disease course and virulence profile, and which are organ-specific in humans. In particular, this study tested seven species of Streptococci that naturally merge with LBO during meals or traditional empirical skin treatments. The role of the present research is to focus on the pathogen’s response in the planktonic and sessile status at different concentrations of LBO and, at the same time, to investigate the composition of the oil prior to, and after, incubation with a set of representative pathogens using high performance liquid chromatography (HPLC) analysis, with the purpose of evaluating a possible interaction between lentisk oil components and bacterial metabolism.

## Materials and Methods

### Lentisk Berry Oil

Lentisk berry oil (LBO) was obtained from *P. lentiscus* fruits from Mediflora^®^ (Pula, Cagliari, Italy) by using ripe berries harvested in winter in the southern Sardinian region (specific weight = 890 Kg/m^3^). After a cycle of light dehydration by air for 20 days, the drupes were cold-pressed following an already described method (Mezni et al., 2014b). The oil was then filtered and stored in stainless steel containers at 20°C until bottling. Prior to use, the sample was centrifuged at 12,000 rpm for 15 min and the supernatant was used for the experiments.

### Bacterial Strains, Media, and Growth Conditions

A set of bacteria/yeasts described in human tissues as pathogens, commensals, or probiotics was used: *S. aureus* ATCC 6538 (American Type Culture Collection), *Staphylococcus hominis* human clinical isolate NC5, *Pseudomonas aeruginosa* ATCC 27853, *Bacillus clausii* isolated from a commercial product, Enterogermina^®^ (Senesi et al., 2001). *Streptococcus agalactiae* human clinical isolate NC2, *Streptococcus intermedius* DSM 20573 (German Collection of Microorganism and cell culture), *Streptococcus mitis* human clinical isolate NC1, *S. mutans* CIP103220 (Collection Institut Pasteur), *Streptococcus pyogenes*, human clinical isolate NC4, *Streptococcus salivarius*, strain K12 isolated from a commercial product (Bactoblis) corresponding to ATCC strain^®^ BAA-1 024 and *S. salivarius* strain M18 isolated from a commercial product (Carioblis^®^). Prior to use, these strains were stored at -80°C in a tube containing Muller Hinton or Schaedler Broth (Microbiol, Uta, Cagliari) with 20% glycerol. Furthermore, three different *Candida* spp. were assayed: *Candida albicans* clinical oral isolate BF01, *Candida glabrata* clinical isolate BF02 and *Candida krusei* clinical isolate BF03. The yeast colonies were identified with an API ID32C AUX system (Biomerieux, St. Louis, MO, United States) and maintained at -80°C in Sabouraud Broth/Glycerol 20% prior to use. For each strain, a growth curve in the corresponding liquid medium plus 10% glycerol was performed. The culture at the mid- lag phase was stored at -80°C prior to use. These bacterial suspensions were used as standardized inoculum to minimize the variation in experimental conditions and ensure reproducible experimental data.

### Agar Diffusion Test

As a first step, an agar diffusion test (Kirby-Bauer) was used to evaluate bacterial resistance/susceptibility (Barry et al., 1979). The protocol was modified due to the high oil density. For each bacterium strain, 15 mL of agarized medium (Microbiol, Uta, Cagliari) at 55°C was added to a 90 mm Petri dish and, prior to agar solidification, four sterile iron rivets, Ø 10 mm diameter and 2 mm thick (Firm, Milan, Italy), were put into the agar mixture and then removed from the medium when it was cold. In these conditions, the wells contained 50 μL of oil. Each strain was inoculated onto the plate surface using a sterile swab with bacterial 5^∗^10^7^ (CFU) standardized inoculum. Three wells were used for the oil testing and two for a negative control. The Petri dishes were incubated in air at 37°C for 24 h for the aerobic strains and in 5% CO_2_ at 37°C for the Streptococcal species. After incubation, the diameter of the possible inhibition alone was measured and the experiment was performed in triplicate. In this experiment, Schaedler agar was used for Streptococci, Muller Hinton agar for aerobic bacteria and Sabouraud agar for yeasts (Microbiol, Uta, Italy). Broth dilution and antibiofilm tests were only used for bacterial genera that showed sensitivity to LBO.

### Broth Dilution Tests, MIC and MBC

The minimum inhibitory concentration and minimum bactericide concentration, MIC and MBC respectively, were performed by the micro-dilution method following CLSI procedures (Mushtaq et al., 2004). The method was executed in sterile Nunc^TM^ Microwell^TM^ 96-well microplates (Thermo Fisher Scientific) and each well was coated with twofold serial dilutions of lentisk oil with Schaedler Broth (from 50 to 0.04%) until a final volume of 0.20 mL for each well. For each strain, 1^∗^10^7^ CFU/mL liquid suspension was prepared from the standardized inoculum in Schaedler broth; 0.02 mL of this suspension were put into each well to obtain a final concentration of 10^6^ CFU/mL. The cultures were incubated at 37°C and 5% CO_2_ for 24 h. To determine the MBC, 150 μl of dilution representing the MIC and two of the more concentrated LBO wells were plated in Schaedler Agar at 37° with 5% CO_2_; after 24 h, the colony-forming units (CFUs) were enumerated. The MBC is the lowest concentration that demonstrates a reduction (such as 99.9%) in CFU/mL when compared to the MIC dilution.

Using the same experimental conditions, we tested the antibacterial activity of serial dilutions of oleic acid, from 500 to 0.5 μg/mL. The MIC and MBC were carried out with oleic acid, 18:1 (n-9) (Sigma-Aldrich, St. Louis, MO, United States).

### Antibiofilm Assay

Lentisk berry oil’s ability to inhibit biofilm formation, MBIC (Subramenium et al., 2015), was evaluated following the modified crystal violet staining protocol described by Montana University Center for Biofilm Engineering^1^. Each *Streptococcus* strain was cultured in triplicate on 96-well microplates with different concentrations of LBO in Schaedler Broth; after 7 days at 37°C with 5% CO_2_, the medium was discarded and the wells were gently washed three times with a 0.9% NaCl solution; then 0.1 mL of a 0.1% of crystal violet solution was added to each well; after 10 min the dye was discarded, followed by three washes with 0.9% NaCl. After an air-drying procedure at 25°C for 15 min, 0.3 mL of 30% acetic acid were added to each well. The plates were read with a microplate reader at 550 nm (SLT-Spectra II, SLT Instruments, Germany). The MBIC represented the lowest concentration showing an absorbance comparable with the negative control (sample without bacteria), in the same concentration series the data showing a Standard deviation (SD) within 10% of the mean value were considered significant.

### Growth Curve Analysis in an Emulsion Bioreactor

**FIGURE 1:**
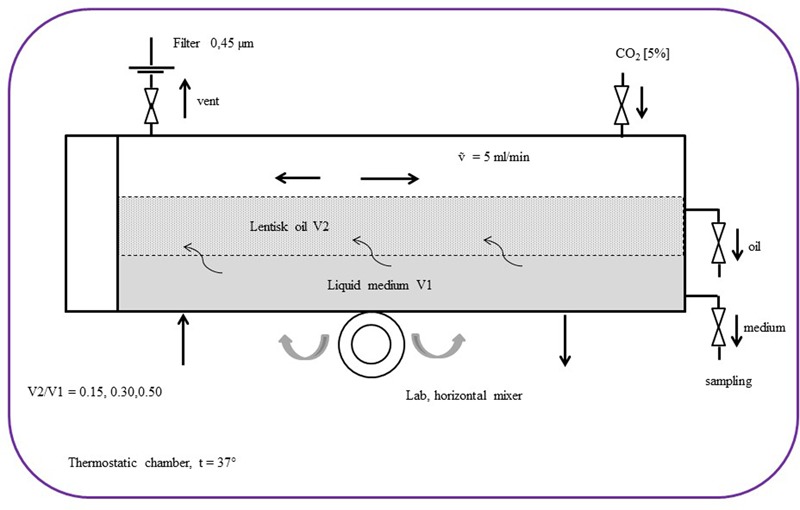
Schematic diagram of the bioreactor set-up with the biphasic emulsion containing lentisk berry oil (V2) and Schaedler broth (V1).

We developed a growth system to increase the bacterium-LBO contact area by means of an emulsion bioreactor (**Figure 1**). The system contained 300 mL of Schaedler Broth/LBO in a biphasic oil/medium phase. Three different LBO concentrations were studied: 15, 30, and 50% LBO respectively. This system produced a number of physical conditions able to promote the growth of the *Streptococcus* strains analyzed in this work; the temperature was maintained at 37°C and the air contained 5% CO_2_. The reactor was kept in continuous agitation by a lab horizontal mixer (Continental Instruments, Italy) with a rotation speed (β) of 15 rpm (Denotti et al., 2009). Single significative LBO resistant strains, such as *S. pyogenes, S. salivarius K12, S. agalactiae* and *S. mutans*, were inoculated with an inoculum of 1^∗^10^6^ CFU/mL.

In these physical conditions, individual oil droplets can be considered as micro incubators containing a considerable number of bacteria on their surface. The adhesion force for the bacterial cell-droplets (ΔE) depends on droplet radius (R), interfacial tension (γ), and the contact angle (𝜃), with: [ΔE = -πR2γ (1-|cos𝜃|)_2_] (Dewey et al., 2014). ΔE also depends on the number of droplets and this is proportional to the bascule rotation rate [ΔE ∝ β], which in this case was 60 cycles/min. Every 30 min, 2 mL of medium (Shaedler Broth) and 0.1 mL of oil fraction were sampled and used for growth curve measurement and for *S. mutans* inhibitory activity detection, respectively. The fatty acid profile in the LBO was measured at T0 and after 8 h using 10 mL of LBO specimens. The growth curves were performed in triplicate by reading the absorbance = 550 nm of the medium by a spectrophotometer (Prixma 5100, Italy, optical path length = 10 mm). For each value series, the max absorbance standard deviation (SD_max_) accepted was ≠20% of the mean value.

### Antibacterial Activity from Bioreactor Oil Fraction

The oral pathogen *S. mutans* was used as a target bacterium to evaluate whether *S. salivarius* K12 or *S. pyogenes* produce antibacterial metabolites from LBO, when cultured in a biphasic oil/medium emulsion. Four emulsion cultures of *S. pyogenes* and *S. salivarius* K12 containing different LBO concentrations (0, 15, 30, or 50%) provided the oil sample aliquots. These were then used at 0 and after 8 h (T0, T8) of incubation to establish the inhibitory activity against *S. mutans*. The antibacterial assay was carried out following the described Kirby-Bauer test by using 50 μL of the bioreactor oil in every well. The growth inhibition zone was assessed after 24 h with 5% CO_2_, and the control (Schaedler broth plus bacterium) was analyzed under the same conditions.

### Fatty Acid Analysis

At (T0) and after 8 h of incubation an aliquot (10 mL) of the bioreactor medium, inoculated with *S. pyogenes*, was used to analyze the fatty acid composition. Fatty acid standards and solvents of the highest purity available were used and purchased from Sigma–Aldrich (Milan, Italy). All the other chemicals used in this study were of analytical grade.

Aliquots (3 mg) of *P. lentiscus* oils dissolved in EtOH (2 mg/mL solutions) were subjected to mild saponification in the dark at room temperature for 14 h (Rosa et al., 2012). The saponifiable fraction with fatty acids was collected, the solvent was evaporated, and a portion of the dried residue was dissolved in CH_3_CN/0.14% CH_3_COOH (v/v). Analyses of fatty acids were carried out with an Agilent Technologies 1100 liquid chromatograph equipped with a diode array (DAD) detector and an Infinity 1260 evaporative light scattering detector (ELSD) (Agilent Technologies, Palo Alto, CA, United States). Unsaturated (detected at 200 nm) and saturated (detected with ELSD) fatty acids were separated with an XDB-C18 Eclipse column (150 mm, 4.6 mm, 3.5 μm Ø particle size) equipped with a Zorbax XDB-C18 Eclipse (12.5 mm 4.6 mm, 5 μm Ø particle size) guard column (Agilent Technologies), with a mobile phase of CH_3_CN/H_2_O/CH_3_COOH (75/25/0.12, v/v/v), at a flow rate of 2.3 mL/min (Rosa et al., 2012). Fatty acid identification was made using standard compounds and the conventional UV spectra, generated with the Agilent OpenLAB Chromatography data system. Calibration curves of all the compounds (correlation coefficients > 0.995) were constructed using standards and were found to be linear for the DAD detector and exponential for ELSD.

#### Analyses of Free Fatty Acids in the Oil

Aliquots of LBO were dissolved in CH_3_CN with 0.14% CH_3_COOH (v/v) to obtain 2 mg/mL solutions. Aliquots of these solutions were directly injected into the HPLC-DAD/ELSD system to determine the quali-quantitative composition of the free fatty acids (FFAs) present in the oils.

### Lipase Assay

To evaluate the lipase activity of non-sensitive native LBO strains (*S. agalactiae, S. pyogenes, S. salivarius*, and *S. mutans*) a lipolytic enzyme test was performed, using the oil/phenol red procedure described by Lee et al. (2015), modified to be used with Streptococci and LBO.

The test was executed in a Petri plate (Ø = 30 mm) containing: phenol red (0.05% w/v), LBO (0.1% v/v) in Schaedler Agar. Each strain was cultured with Schaedler Broth/LBO (50%) in a shake emulsion culture at 37°C for 24 h. After centrifugation at 6000 rpm for 10 min, the bacteria were recovered and re-suspended in a 0.9% saline solution. 0.1 mL of 0.5 McFarland standard suspension corresponding to 10^8^ CFU/mL was inoculated onto the center of the plate containing a fissure obtained through the rivet method. After 24 h of incubation at 37°C and 5%, the CO_2_ yellow alone diameter was measured (ø mm) and reported as a semi-quantitative lipase assay. Lipase activity was calculated with the subsequent formula:

[Lipase act = Ø mm _Lbo_ – Ø mm _control_]

where: Ø mm _Lbo_ = the diameter of the yellow alone around the fissure in the medium with LBO and Ø mm _control_ was the diameter in the same medium without LBO.

### Comparative Sequence Analysis of MCRA Proteins

We analyzed and compared *in silico* 31 different Myosin cross reactive antigen sequences (MCRA) for *Streptococcus* spp., available in the UniProt data bank and in the NCBI Protein database. The alignments of multiple sequences were analyzed using the Clustal Omega program^2^. The phylogenetic tree was generated using GeneBee-NET software and Phylogeny.fr^3^ according to a neighbor joining algorithm (Brodskii et al., 1995). **Table 1** shows the MCRA accession number relative to the Streptococci used in this work.

**Table 1:** Bacterial strain used in this work and analyzed for MCRA protein phylogenesis.

Strain	LBO susceptibility group	MCRA accession number	Reference
*S. agalactiae*	II	OVE42222, ASI66524, KUH49693	
*S. mutans*	II	ARS61960, GAW69524, WP_024786556	Zheng et al., 2014
*S. pyogenes*	II	ANP29019, OAC73215, OAC78672	McShan et al., 2008
*S. salivarius*	I	ARI58907, CCB94037, CCB94037, EGX30691	

### Statistical Analysis

Evaluation of the statistical significance of differences was performed using Pearson’s chi-square test for antimicrobial activity experiments and one-way analysis of variance (one-way ANOVA), followed by the Bonferroni multiple comparison test for chemical data, using GraphPad InStat software (San Diego, CA, United States).

## Results

### Antimicrobial Assays

An initial evaluation using Kirby-Bauer analysis showed that yeasts, *Candida* spp. and Gram-negative bacteria did not demonstrate appreciable sensitivity to this extract. The same also held true for *S. aureus, S. hominis* and *B. clausii*, but an unusual antimicrobial profile was observed within the *Streptococcus* genus. In fact, native LBO resulted active against two different strains: *S. mitis* and *S. intermedius* (inhibition diameter 12≠1 and 15≠1 mm respectively), while it appeared to be inactive against *S. salivarius* and other Streptococci. This first experiment suggested a possible differential activity within *Streptococcus* spp. and, from this point on, we investigated their interaction with LBO. The antimicrobial-anti biofilm analysis evaluated in microplates through the use of a liquid medium partially reflected the results suggested by the Kirby-Bauer procedure with substantial differences amongst the native LBO non-sensitive strains. In fact, by comparing different LBO concentrations with the studied Streptococci, we were able to observe three types of “sensitivity motifs” (**Table 2**). These values were thus in accordance with the MIC values obtained with oleic acid and were correlated with the lipase–hydrolase activity, as described later. The *S. salivarius* K12 and M18 probiotic strains confirmed their non-sensitivity to lentisk, group I (MIC and MBIC > 50%). Other Streptococci followed two trends:

**Table 2:** Sensitivity motifs of lentisk oil versus analyzed Streptococci in comparison with lipase activity.

Bacterial strains	MIC	MBC	MBIC	MIC	LBO susceptibility group		Lipase activity
	Lentisk oil %			Oleic acid μg/mL			ø mm
*S. salivarius* K12 and M18	>50	>50	>50	>500	(I)	Non-sensitive	5 ± 1
*S. pyogenes, S. agalactiae, S. mutans*	50	50	25-3	250-5	(II)	Partially sensitive	7–5 ± 1
*S. intermedius, S. mitis*	6-3	50	<4	0,5	(III)	Sensitive	nd

*nd, non- detectable.*

(i) High MIC value [50%] and a biofilm structure observable from [25–3%] in LBO dilution, group II.

(ii) Very sensitive strains characterized by low LBO MICs [6–3%] and low LBO MBIC [<4%], group III.

### Lipase Assay

By comparing the diffusion alone obtained with the studied Streptococci, great activity was observed after 12–24 h with *S. pyogenes* (Ø = 7 ± 1 mm diffusion), while the other Streptococci showed a low activity (Ø = 5 ± 1 mm). This was why we chose to study the HPLC profile of FFAs with *S. pyogenes* (**Table 2**).

### Streptococci Behavior in the Biphasic Emulsion Bioreactor

**FIGURE 2:**
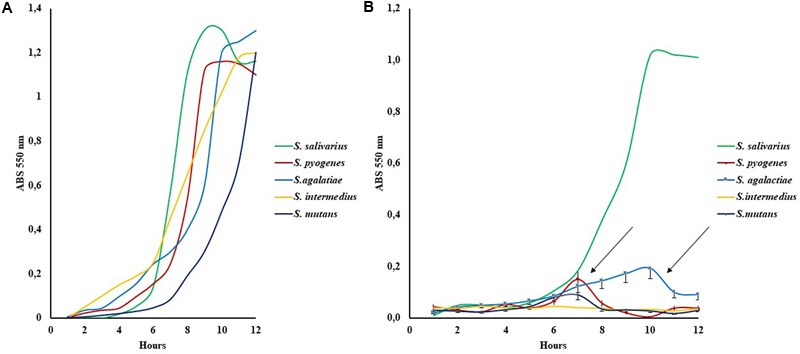
Growth curves of five indicative *Streptococcus strains* in Schaedler broth **(A)** and with Schaedler broth LBO 50% **(B).** The arrows show the bell curves observed in Group II susceptibility strains (*S. pyogenes, S. mutans, S. agalactiae*), SD % ranged from 15 to 20% of the mean value.

The presence of LBO in the bioreactor medium imposed a different growth trend as shown in **Figure 2**. Group I, *S. salivarius* K12 presented a classic curve comparable to growth without oil, whereas the partially sensitive LBO group II, *S. pyogenes, S. agalactiae, S. mutans*, showed an interesting trend: the observed “bell curve” could indicate possible inhibition after an initial growth, which suggests that secondary metabolites may act as antimicrobials. These curves were designed by using the medium value of three experiments and the max Standard Deviation observed (SD_max_) was = 20%, from the absorbance medium value. In these conditions, the first point of decrease in growth on the curve was observed at 7/8 h for *S. pyogenes* and *S. mutans*, while it occurred at 9–10 h for *S. agalactiae*, indicating a possible representative concentration of these hypothetical antibacterial metabolites (**Figure 2**).

### Fatty Acid Analysis

**FIGURE 3:**
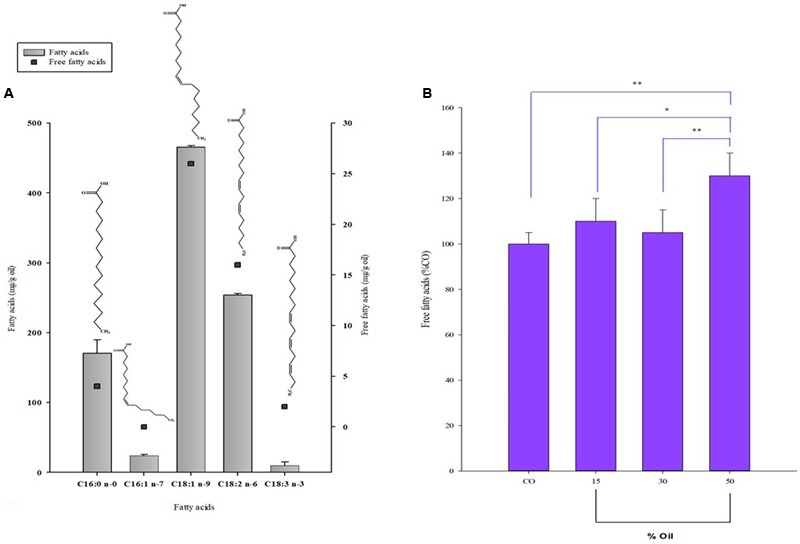
Fatty acid composition of LBO before and after *Streptococcus* growth. **(A)** Composition of the main fatty acids (FA) right Y axis and free fatty acids (FFA) left y axis (mg/g oil) of *Pistacia lentiscus* berry oil (LBO) detected by HPLC analysis. **(B)** Total amount of free fatty acids (Σ of free 16:0, 18:1 n-9, 18:2 n-6, and 18:3 n-3) reported as % with respect to control, native LBO (CO), detected in the emulsion samples after 8 h incubation with *S. pyogenes.*^∗∗^*P* < 0.01 vs. CO; ^∗^*P* < 0.05 vs. 15% [LBO]; ^∗∗^*P* < 0.01 vs. 30% [LBO].

Quali-quantitative information on the individual fatty acids that compose the lipid classes of *P. lentiscus* oil used in the growth experiment with *S. pyogenes* was obtained by HPLC-DAD/ELSD analyses after lipid saponification. The values of the main saturated and unsaturated fatty acids (UFAs) (expressed as mg/g of oil) of *P. lentiscus* oil are reported in **Figure 3A**. The oil was characterized by a high level of oleic acid C18:1 n-9 (465.5 ± 2.0 mg/g of oil extract), linoleic acid C18:2 n-6 (254.2 ± 5.5 mg/g of oil extract), and palmitic acid C16:0 (170.6 ± 19.5 mg/g of oil extract), with a low amount of α-linolenic (C18:3 n-3) and palmitoleic (C16:1 n-7) acids. The oil also showed a detectable amount of FFAs, and values of the free form in the range of 5–12% were measured using HPLC for the UFAs. After incubation with *S. pyogenes*, LBO added at different amounts (15, 30, and 50%) to the culture medium, was separated and subjected to lipid saponification for the determination of the total fatty acid profile and directly analyzed for the quantification of FFA. No significant differences were observed in the total fatty acid profile of oil samples after microorganism incubation as compared to the control oil (LBO). However, a significant increase (133% of CO, *P* < 0.01) in FFA content (as the sum of free C16:0, C18:1 n-9, C18:2 n-6, and C18:3 n-3) was observed in 50% of oil samples *versus* the *native* oil control (**Figure 3B**).

### Antibacterial Activity of the Oil Fraction Collected from the Bioreactor

**FIGURE 4:**
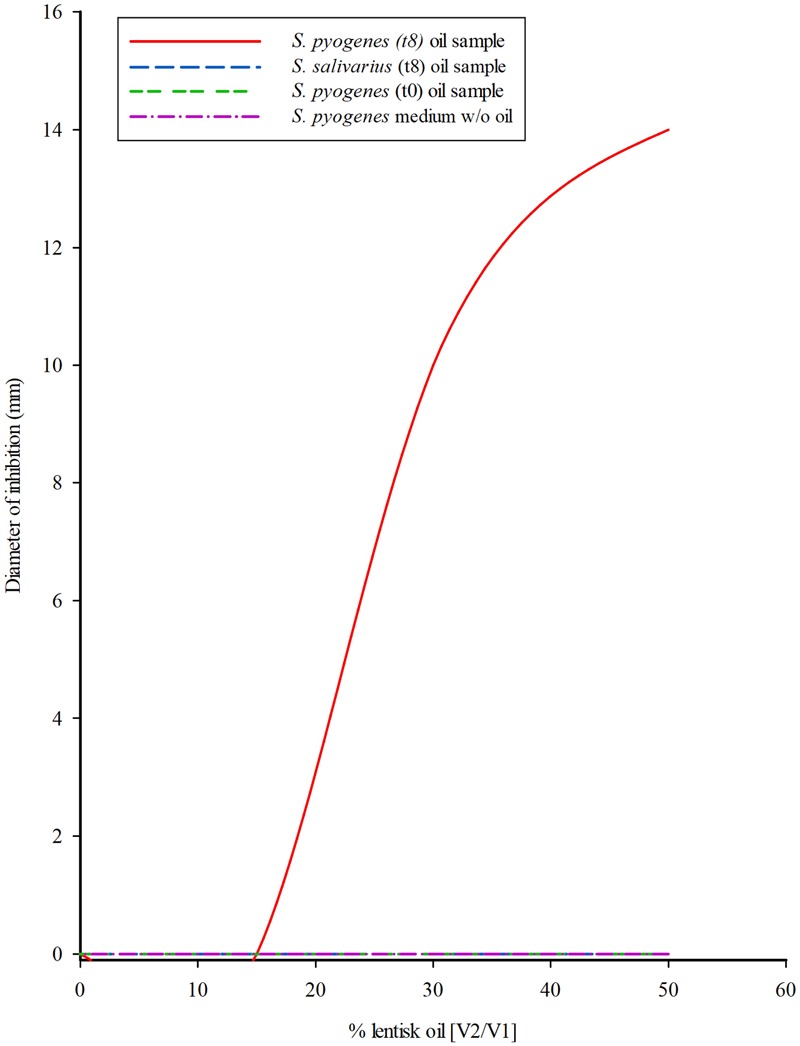
*Streptococcus mutans* growth inhibition from LBO bioreactor fraction. Tendency curve of *S. mutans* inhibition by the oil sampled from the 50% LBO/broth (V2/V1) biphasic cultures of *S. pyogenes* and *S. salivarius* K12, in the bioreactor system at onset (T0) and after 8 h (T8) incubation. The experiment was performed at 37°C according to the Kirby-Bauer Agar diffusion test using 0.05 mL of oil. Data were collected after 24 h incubation. The third level polynomial curve obtained with three different measures showed a regression coefficient *R*^2^ = 1.

*Streptococcus mutans* inhibition was observed with oil sampled after *S. pyogenes* culture and shows an approximately third level polynomial curve function (*R*^2^ = 1) (**Figure 4**). The relationship between the percentage of lentisk oil and the diameter (mm) of the inhibition zone could be represented as follows: [Y = -7^∗^10^-4^X^3^+5^∗^10^-2^X^2^-0.6^∗^X+3^∗^10^-12^], where: Y = medium of diameter inhibition (Ø mm) and x represents the percentage of lentisk oil in the bioreactor. Neither the respective liquid medium (Shaedler broth) fraction, nor any of the LBOs coming from *S. salivarius* showed an appreciable antibacterial activity. The represented curve was performed by using the values of three experiments.

### Minimum Inhibitory Concentration of Oleic Acid

We observed the complete inhibition of *S. intermedius* and *S. mitis* (group III) at a low concentration of oleic acid (0.5 μg/mL), while the *S. salivarius* K12 (group I) showed a MIC > 500 μg/mL. For partially sensitive strains, group II, i.e., *S. pyogenes*, the MIC values were comprised between 250 and 0.5 μg/mL. These results indicate a striking difference in sensitivity to oleic acid amongst the analyzed Streptococci at concentration ranges of oleic acid from 500 to 0.5 μg/mL, as described by other authors (Speert et al., 1979; Speert and Wannamaker, 1980) (**Table 2**).

### Comparative Sequence Analysis of MCRA Protein

**FIGURE 5:**
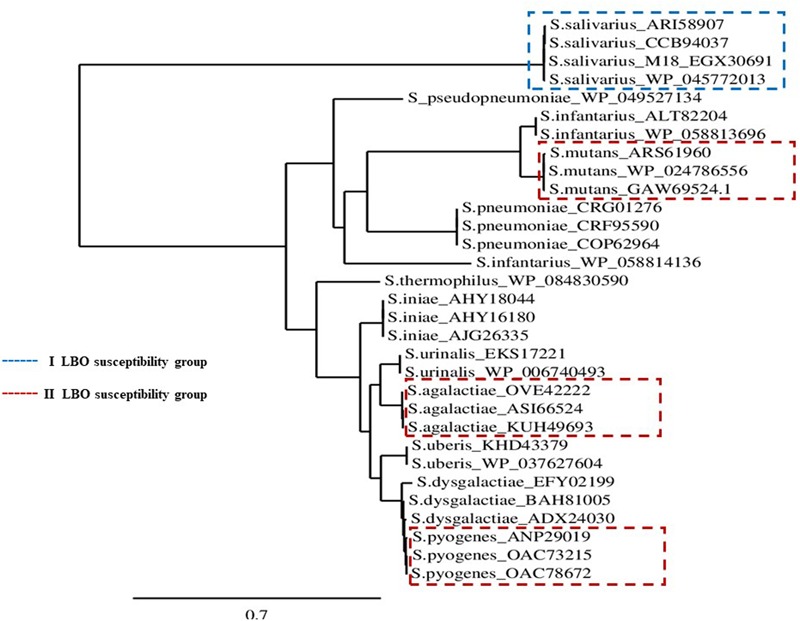
MCRA protein Phylogenetic Tree. Scenario of the MCRA protein phylogenies for different *Streptococcus* spp. with respective GenBank accession numbers, available in the NCBI protein database.

All MCRA protein sequences relative to different *S. salivarius* strains, extracted from the NCBI Data Bank, were comprised in the same identity group. At the same time, this group showed a considerable aminoacidic identity distance in comparison with other protein sequences published for other Streptococci (**Figure 5**).

## Discussion

The quest for new plant-based antibacterial drugs is now considered an important field in antimicrobial research. The increasing frequency of new drug-resistant bacteria and the high costs required for new patented chemically derived drugs, especially in developing countries (Fair and Tor, 2014) have placed particular emphasis on edible and non-edible fractions of antimicrobial drug derived from vegetables. In these compounds, secondary metabolites such as phenol tannins, terpenoids, alkaloids, flavonoids, linear peptides, etc. may possess specific biological activities against many pathogenic microorganisms. Chemical extraction processes from plants, e.g., those using aqueous or organic solvents or supercritical fluid extraction, may sometimes influence not only the concentration of the active compounds, but also the presence of toxic metabolites (Scur et al., 2016; Silva et al., 2016; Arbia et al., 2017; Khan et al., 2017; Petrovic et al., 2017; Rosas-Burgos et al., 2017; Thielmann et al., 2017; Zbikowska et al., 2017). The use of the edible part of plants could minimize this problem, especially when this product has been used for 100s of years in human alimentation, as is the case for lentisk oil.

Most of the current studies on lentisk antibacterial activity use the non-edible part of this plant, such as essential oil from mastic branches and leaves (Mharti et al., 2011; Mezni et al., 2016; Piras et al., 2017). On the contrary, very little is known about LBO, namely the edible part of lentisk. In these reports, several variables seem to be determinant for the quality of the antimicrobial profile of the lentisk extracts. Among these, the characteristics of the geographical region such as land composition, climatic *status*, harvest date, etc. play an important role.

The plant’s location, climatic conditions and soil composition could explain the fact that the Sardinian LBO activity against *S. aureus* described in our study was not found in previous studies performed using Tunisian LBO (Mezni et al., 2014a, 2016).

Many evidences suggest that LBO contains different classes of substances with antimicrobial effects: phenols and free unsaturated-saturated fatty acids (UFAs-SFAs). Interestingly, lentisk fruit oil contains a prevalent concentration of UFAs (mean ratio of saturated/unsaturated fatty acids of 0.4) and a considerable component of these are represented by esters of these FFAs (Trabelsi et al., 2015; Mezni et al., 2016).

Our results suggest that LBO contains two groups of antibacterial substances: (i) active native antibacterial components and (ii) potential antimicrobial components activated after bacterial uptake [such as secondary metabolites from fatty acid esters (FAEs)].

The first class with an initial antibacterial activity, which is probably very active in *S. intermedius* and *S. mitis*, is represented by phenols and some FFAs. In fact, these strains showed a low value of MIC oleic acid, group III (**Table 2**).

The second class, represented by esters lacking any native antibacterial activity, seems to be the substrate for new FFAs formed by bacterial metabolism. This was why our study analyzed the role of lipases (esterase–hydratases) in *Streptococcus* spp., as one of the main bases of LBO selectivity. This hypothesis was supported by all the results obtained from lipasic activity in Streptococci (**Table 2**); from the growth curves in the medium containing lentisk oil (**Figure 2**); from the LBO chemical composition before and after bacterial growth (**Figures 3A,B**) and from *S. mutans* growth inhibition in the LBO bioreactor fraction (**Figure 4**).

In Group II Streptococci, which are not sensitive to native LBO, our experimental data suggest the presence of secondary inhibiting factors during their growth with this alimentary substance. In fact, for example, *S. pyogenes* showed a sudden growth regression after 7–8 h (**Figure 2**). At this time, the oil fraction present in the growth medium contains an increased concentration of newly synthesized FFAs (**Figure 3B**). When the same 7–8-h *S. pyogenes* growth medium was used with *S. mutans*, this no-sensitive strain became proportionally sensitive to LBO % (**Figure 4**).

In the context of Streptococci fatty acid metabolism, the myosin cross-reactive antigen (MCRA) protein is the most studied enzyme. It represents a family of proteins that are present in a wide range of bacteria, with a main hydratase activity. The role of MCRA seems to be determinant in oleic acid detoxification (Volkov et al., 2010) hydrolyzing triacylglycerols in a lipid–water interface (Nardini et al., 2000). Some of these proteins are considered as important pathogen determinants in various microbial classes (Volkov et al., 2010) and have also been used in the detoxification of oil-base pollutants (Jeganathan et al., 2007). Interestingly, deletion of the MCRA hydratase gene caused a twofold decrease in inhibitory activity against oleic acid (MIC) and plays a role in the virulence of *S. pyogenes* (Volkov et al., 2010). The phylogenetic analysis of the protein sequences performed in our study suggests that significant MCRA polymorphisms could be associated to differences in lipase–hydrolase activity (**Figure 5**).

**FIGURE 6:**
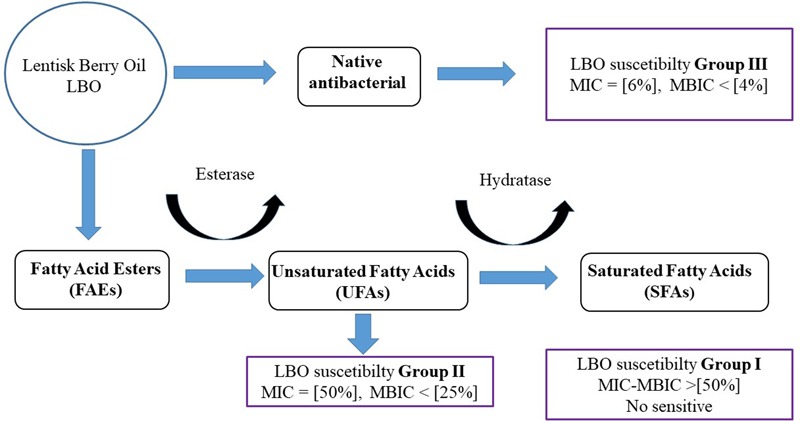
Representative hypothesis of LBO differential activity with different *Streptococcus* spp. This figure represents the hypothesis on LBO function against a Streptococcal population. The final result could be due to different factors: (i) UFA antimicrobial resistance and (ii) esterase hydratase profile/activity in each strain. The initial concentration of phenols, FFA and esters in native LBO could change the susceptibility pattern between Streptococci Groups II and III.

The resistance of the *S. salivarius* probiotic strain to native LBO inhibition and at oleic acid (**Table 2**) could partly be justified by its MCRA profile (**Figure 5**). In this framework, the final antibacterial result could depend on three biological processes: (i) MIC profile *versus* native antibacterial compounds in the LBO, (ii) the speed of new UFA production from lentisk esters and (ii) the speed of UFA removal by hydratase activity (**Figure 6**). The possibility that *Streptococcus* spp., such as groups I and II, could be able to modulate the antibacterial profile of LBO through their fatty acid metabolic pathway is also supported by different results obtained in other studies on the antibacterial role of UFAs in Gram-positive bacteria (Kabara et al., 1972, 1973; Carballeira et al., 1998; Carballeira and Pagan, 2001).

Further experiments are necessary to strengthen this hypothesis, including, (i) *in vitro* characterisation of the complete Streptococcal lipases, (ii) MCRA enzyme kinetics in different strains, and (iii) lipase/hydratase gene expression during Streptococci growth in LBO, which could all add new details about LBO antimicrobial activity.

The results of this study could be important for an antimicrobial preventive clinical approach. For example, this plant-based antibacterial drug could be used as a coadjutant in non-surgical therapy against periodontal diseases or after dental implant placement. In this case, these substances might be able to contrast the formation of pathogenic biofilm, where the role of oral Streptococci appears to be crucial (Pita et al., 2015).

## Conclusion

For the first time ever, the results obtained in this study underline a possible selective antibacterial activity of an alimentary oil obtained from *P. lentiscus*. In the human microbiota era, new investigation strategies, similar to the ones performed in this study, are necessary in order to evaluate plant antimicrobial activity, since a traditional laboratory approach may underestimate some biological effects.

## Author Contributions

GO: designed the study, primary author of the manuscript. CD, AM, and GD: maintained the bacterial strains and the lentisk oil extracts, prepared cultures and performed the antibacterial experiments. GO and GP: performed the bioreactor and lipase test. AR and ET chemical analysis of lentisk oil by HPLC procedure: AR, PR, FC, and PC: assisted in writing the paper and critical analysis of the manuscript. GO and PC: performed the bioinformatic analysis. All authors read and approved this manuscript.

## Conflict of Interest Statement

The authors declare that the research was conducted in the absence of any commercial or financial relationships that could be construed as a potential conflict of interest. The reviewer AC and handling Editor declared their shared affiliation.

## Footnotes

1 http://www.biofilm.montana.edu/

2 http://www.ebi.ac.uk/Tools/msa/clustalo/

3 http://www.phylogeny.fr/index.cgi
